# Is a Second Transcranial Doppler Study Needed to Confirm Neurocirculatory Arrest?

**DOI:** 10.1007/s12028-025-02241-0

**Published:** 2025-03-25

**Authors:** Tiffany Eatz, Yosdely Cabrera, Frank Cabrera, Mohan Kottapally, Amedeo Merenda, Ayham Alkhachroum, Jose G. Romano, Sebastian Koch

**Affiliations:** Department of Neurology, The University of Miami Health System, 1400 NW 12th Ave, Miami, FL 33136 USA

**Keywords:** Transcranial, Doppler, Ultrasound, Brain death, Cessation, Flow

## Abstract

**Background:**

In determining brain death, transcranial Doppler (TCD) is one of the recommended ancillary tests when clinical examinations and apnea tests are contraindicated. The American Academy of Neurology 2023 guideline updates and 2020 World Brain Death Project advise conducting two TCDs 30 min apart to diagnose neurocirculatory arrest. Our study aimed to evaluate whether a second TCD is necessary when the first TCD shows neurocirculatory arrest (no flow, oscillating flow, or systolic spikes).

**Methods:**

We conducted a single-center retrospective analysis of patients admitted to intensive care units from January 1, 2021, to February 1, 2025, at a community-based academic hospital. We included patients whose first study showed neurocirculatory arrest and who subsequently underwent a confirmatory TCD at least 30 min apart. A total of 48 patients were included in our final analysis. We compared the findings of the first TCD study with those of the second study and noted any differences.

**Results:**

In all 48 patients (100%), the second TCD confirmed the findings of the first TCD. Of these 48 patients, 44 patients (91.7%) had the same flow pattern on repeat TCD examination and 4 patients’ (8.30%) TCDs showed different flow patterns, although still consistent with neurocirculatory arrest. Of the 44 patients with the same flow patterns found on first and repeat TCD examinations, 18 patients (40.9%) had both TCDs demonstrate brief systolic spikes; three patients (6.80%) had both TCDs demonstrate brief systolic spikes and oscillating flow; eight patients (18.2%) had both TCDs demonstrate no flow; seven patients (15.9%) had both TCDs demonstrate no flow and brief systolic spikes; one patient (2.30%) had both TCDs demonstrate no flow, brief systolic spikes, and oscillating flow; and, lastly, seven patients (15.9%) had both TCDs demonstrate oscillating flow.

**Conclusions:**

We found that requiring two sequential TCD examinations to confirm neurocirculatory arrest may be unnecessary when the first TCD shows neurocirculatory arrest. Further investigation and studies such as ours in larger populations are warranted.

## Introduction

There are global discrepancies regarding the concept and criteria of brain death (BD) or death by neurological criteria [1]. In the determination of BD, transcranial Doppler (TCD) is one of the ancillary tests recommended when a clinical examination and an apnea test cannot be fully completed due to facial, skull, and cervical cord injuries or medical contraindications. The American Academy of Neurology 2023 guideline updates and the 2020 World Brain Death Project advise care providers to conduct two TCDs 30 min apart to diagnose neurocirculatory arrest [2]. However, we are not aware of any evidence to support the recommendation for a second TCD.

Neurocirculatory arrest is diagnosed through TCD by the presence of (1) no flow, (2) oscillating flow, or (3) brief systolic spikes in the proximal large intracranial arteries (internal carotid arteries, basilar and vertebral arterie, and anterior, middle, and posterior cerebral arteries) [2, 3].

In this study, we aimed to determine whether a second TCD after a primary TCD demonstrating neurocirculatory arrest is necessary or potentially redundant.

## Methods

We conducted an institutional review board–approved single-center retrospective analysis of patients admitted to intensive care units from January 1, 2021, to February 1, 2025 at an academic tertiary medical center who were evaluated for neurocirculatory arrest with TCD. Confirmation of neurocirculatory arrest was arrived at by TCD demonstration of at least one of the following: no flow, brief systolic spikes, or oscillatory flow. Inclusion criteria for final analysis were as follows: (1) patients underwent two sequential TCD at least 30 minutes apart to evaluate for cerebral blood flow, with (2) the first TCD showing neurocirculatory arrest. Overall, 82 patients had a TCD for BD determination because an apnea test was not possible during the study period. We primarily excluded 17 patients because their first TCD did not show neurocirculatory arrest. This exclusion yielded a total of 65 patients included in our initial comparative analysis. We then excluded 17 of these 65 individuals with BD from our final diagnostic analysis because they only had one TCD. This resulted in 48 patients included in our final analyses. The following were also acquired from electronic medical records when available: demographics (age, sex, race), admission data (admit date, admit time), admission clinic details (National Institutes of Health Stroke Scale and Glasgow Coma Scale [GCS], type of brain injury and location), TCD records (date, time, and finding of each TCD, indication for initial TCD, time between two TCDs, whether the same technician executed both studies, and whether the same physician interpreted both studies), other ancillary studies (time, date, and findings), and clinical outcome data (date of declared BD, cause of BD, and organ donation procurement). Indications for initial TCD included hemodynamic instability, cervical cord injury, facial or skull deformity or injury, hypercapnia, respiratory failure, and pneumothorax. Outcomes were measured by assessing whether a repeat TCD also demonstrated BD after a first TCD revealed BD, both through the presence of at least one of the following: no flow, brief systolic spikes, or oscillatory flow.

### TCD Technique Protocol

Transcranial Doppler studies were performed by one of two certified ultrasound technologists (FC or YC) using a 2-MHz pulsed Doppler ultrasonography (Sonara; Natus Medical, Middleton, WI). TCD findings were interpreted by a neurosonology-certified vascular neurologist. Following a standard bilateral insonation protocol through the temporal acoustic window, the anterior, posterior, and middle cerebral arteries as well as terminal internal carotid arteries were evaluated for cerebral flow throughout their course at 2-mm intervals. Similarly, the intracranial vertebral and basilar arteries were insonated through a transforaminal approach, and the flow patterns were digitally recorded for review.

The following flow patterns on TCD were regarded as consistent with a diagnosis of neurocirculatory arrest: (1) brief systolic forward flow or systolic spikes and diastolic reverse flow, (2) brief systolic forward flow or systolic spikes and no diastolic flow, or (3) no demonstratable flow in a patient in whom flow had been clearly documented in a previous TCD examination. The above TCD findings were only accepted as confirmatory of BD when they were found bilaterally in the middle cerebral, intracranial carotid, and basilar arteries.

### Statistical Analysis

Patients with BD who underwent two TCDs were analyzed by comparing the findings of the second TCD to the first to see if they were the same or different. The location of cerebral injury, indication for TCD, clinical scores on arrival (GCS and National Institutes of Health Stroke Scale), and ancillary tests were investigated. Analyses were descriptive in nature, performed in R version 4.1.1 [4]. To minimize bias of excluding patients with BD with only one TCD, we preliminarily compared those patients who underwent two TCDs (*n* = 48 patients) with those who only had one TCD demonstrating BD (*n* = 17). Demographics were compared to assess whether there was a statistically significant difference in means for age and in ratios for race and sex between the included and secondarily excluded patients with BD. Two-sample *t*-tests and Z-tests were performed with a statistical significance set at a threshold of *p* < 0.05.

## Results

The mean age of the 48 patients included in our analysis was 48.0 years old (standard deviation 17.5 years). Women comprised 35% of the included patients, and men comprised 65%. The included patients were 54% white, 31% black, 2% mixed black and white, and 13% racially unidentified as well as 33% Hispanic, 61% non-Hispanic, and 6% with unknown Hispanic status. Demographic and medical characteristics of the included 48 patients are depicted in Table 1.

**Table 1:** Characteristics of included patients with brain death who underwent two TCDs (*n* = 48 patients)

	Patients (*n*)	Patients (%)
Race and ethnicity		
White	26	54.2
Black	15	31.2
Mixed black and white	1	2.10
Unknown	6	12.5
Hispanic	16	33.3
Non-Hispanic	29	60.4
Unknown Hispanic	3	6.30
Sex		
Female	17	35.4
Male	31	64.6
TCD reading		
Same 1st and 2nd	48	100
(NA vs. non-NA)		
Different 1st and 2nd	0	0.00
Brain injury type		
Traumatic hemorrhage	17	35.4
Nontraumatic hemorrhage	11	22.9
Hypoxic and anoxic	17	35.4
Herniation	11	22.9
Cervical spinal fracture	1	2.08
Brain injury location		
Right hemisphere	4	8.33
Left hemisphere	9	18.8
Bilateral	33	68.8
Cerebellum	4	8.33
TCD indication		
Hemodynamic instability	31	64.6
Cervical cord injury	1	2.08
Facial deformity	1	2.08
Hypercapnia	2	4.16
Respiratory failure	12	25.0
Pneumothorax	1	2.08
GCS (mean 3.8, SD 2.4)	27	56.3
NIHSS (mean 16.3, SD 11.9)	4	8.30
Clinical scores upon arrival		
NMR spectroscopy	10	20.8
Ancillary tests		
VEEG	5	10.4
CTA head and neck	2	4.20

*CTA*, Computed tomography angiography, *GCS*, Glasgow Coma Scale, *NA*, Neurocirculatory arrest, *NIHSS*, National Institutes of Health Stroke Score, *NMR*, Nuclear magnetic spectroscopy, *SD*, Standard deviation, *TCD*, Transcranial Doppler, *VEEG*, Video electroencephalogram

Of these 48 patients, 17 (35.4%) experienced a traumatic brain injury. Eleven patients (22.9%) presented with nontraumatic intracerebral hemorrhage, 17 (35.4%) presented with hypoxic and anoxic brain injury, and 11 (22.9%) presented with herniation secondary to mass effect. Of these 11 patients with herniation secondary to mass effect, 6 (54.5%) patients were primarily incited by traumatic brain injury, 3 (27.3%) patients were primarily incited by anoxic injury, one patient (9.10%) was primarily incited by subarachnoid hemorrhage, and one by pyogenic ventriculitis. Four patients (8.33%) had injury to the right hemisphere, 9 (18.8%) patients had injury to the left, 33 (68.8%) patients had injury bilaterally, and 4 (8.33%) patients had injury to the cerebellum (some injury sites overlapped).

TCD indications included 31 patients (64.6%) with hemodynamic instability, 1 patient (2.08%) with facial deformity or injury, 1 patient (2.08%) with cervical spinal cord injury, 2 patients (4.16%) with hypercapnia, 12 patients (25.0%) with respiratory failure, and 1 patient (2.08%) with pneumothorax. Indications were overlapping for some patients. The median GCS on arrival was 3 (interquartile range 0 [3]), which was reported in 28 (58.3%) of the 48 patients. Lastly, other ancillary tests were conducted, including nuclear magnetic resonance spectroscopy in ten patients (20.8%), video electroencephalograph in five patients (10.4%), and computed tomography angiography (CTA) head and neck in two patients (4.20%), although the American Academy of Neurology does not recognize CTA as an accepted ancillary test in the diagnosis of BD. However, other countries do. All these ancillary examinations (100%) were consistent with neurocirculatory arrest.

All (100%) of the repeat studies were executed by the same technician and interpreted by the same physician as was its respective first study. Of the 48 patients who underwent two TCDs with the first demonstrating neurocirculatory arrest, 48 patients (100%) demonstrated neurocirculatory arrest (no flow, brief spikes, or oscillatory flow) on second TCD. No patients (0.00%) had a second TCD that showed cerebral blood flow. Of the 48 patients with reported distinguishable TCD patterns, 44 patients (91.7%) demonstrated the same flow pattern on repeat TCD examination, and four patients’ (8.30%) TCDs showed different flow patterns, although still consistent with neurocirculatory arrest. Of the four patients with a different flow pattern on first and second TCD examination, one patient’s first study demonstrated brief systolic spikes and no basilar flow, whereas their second study showed no middle cerebral artery flow and oscillating flow in the basilar artery. The second patient’s first TCD showed brief systolic spikes and their subsequent study demonstrated no cerebral flow. Both the third and fourth patients’ first TCDs demonstrated no intracerebral flow; their second study showed a mixture of no flow and oscillating flow. Of the 44 patients with the same flow patterns found on first and repeat TCD examinations, 18 patients (40.9%) had both TCDs demonstrate brief systolic spikes; 3 patients (6.80%) with brief systolic spikes and oscillating flow; 8 patients (18.2%) with no flow; 7 patients (15.9%) with no flow and brief systolic spikes; 1 patient (2.30%) with no flow, brief systolic spikes, and oscillating flow; and, lastly, 7 patients (15.9%) with oscillating flow.

We compared the characteristics of the 17 excluded patients who only had one TCD consistent with neurocirculatory arrest to assess for any selection bias. The mean age was 44.7 years (standard deviation 16.8 years), with 76% being men and 24% being women. In this group, 76% of the patients were white and 24% were black; 53% were Hispanic, 41% were non-Hispanic, and 6% were with unknown Hispanic status. There was no statistically significant difference in means of age (*p* = 0.5022) or in ratios for race and sex (*p* range = 0.9113–0.9955 and 0.9412–0.9593, respectively) (Table 2).

**Table 2:** Patients with brain death with two TCDs (*n* = 48) vs. those with only one TCD (*n* = 17); (total *N* = 65 patients)

Parameter	Significantly different? (yes or no)^a^	Difference^b^	*p* value
Mean age (yr)	No	3.300	0.5022
Race and ethnicity			
White	No	0.223	0.9192
Black	No	0.077	0.9600
Mixed black and white	No	0.021	0.9527
Hispanic	No	0.196	0.9113
Non-Hispanic	No	0.192	0.9275
Unknown Hispanic	No	0.004	0.9955
Sex			
Female	No	0.119	0.9412
Male	No	0.119	0.9593

The demographic discrepancy of the two population subsets (patients with brain death with 2 TCDs and those with only 1 TCD) are statistically insignificant; therefore, these two cohorts are demographically comparable. TCD, transcranial Doppler

^a^Statistically significance set at a threshold of *p* < 0.05

^b^For age, difference indicates differences in means; for race and sex, difference indicates difference in ratios

## Discussion

The results of this study illustrated that when the findings of an initial TCD are consistent with neurocirculatory arrest, those of a repeat TCD are as well. This notion begs the question whether a second TCD is necessary in the diagnosis of BD if the first TCD demonstrates no flow, oscillatory flow, or brief systolic spikes. A second TCD may create unnecessary burden on the health care system, requiring equipment, time, technicians, and physicians to perform and analyze the examination. This allotment may take away resources and time from other clinical needs. Additionally, delay in official declaration of BD may contribute to loss of preservation of viable organs for donation due to hemodynamic deterioration in the time between the first and second TCD [5, 6].

A single TCD has been shown to be a highly accurate ancillary test for BD confirmation, with a sensitivity of 90% and specificity of 98% [2, 7]. We are not aware of any other studies evaluating the necessity of a second TCD. To our knowledge, there are presently no recommendations to repeat other ancillary tests, such as nuclear magnetic resonance spectroscopy, four-vessel catheter angiography, head and neck CTA, magnetic resonance angiography, and video electroencephalograph.

BD diagnostic protocol differs globally. Cerebral blood flow investigation is mandatory in 18% of European countries and optional or conditional in 82% [8]. Some countries other than the United States do not require a repeat TCD. For example, Brazil, Germany, and China, among others, do not specify a requirement of two TCD examinations consistent with neurocirculatory arrest to confirm the diagnosis [9–12].

Contrarily, some investigators propose that the purpose of performing a second TCD is to increase diagnostic certainty, thereby preventing misdiagnosis [13–15]. A study by Dosemeci et al. [16] found that repeat TCDs increase the test’s sensitivity. They reported a sensitivity and specificity of a first TCD examination to be 70.5% and 97.4%, respectively [16]. The sensitivity in their study reached 100% by the fourth TCD [16]. However, overall, these authors did not investigate whether a primarily neurocirculatory arrest study reverted to a non-BD pattern on subsequent TCD examination. All their analyzed TCDs displayed an increase in sensitivity, with progression to patterns consistent with neurocirculatory arrest on serial studies, the latter of which is consistent with our findings. Notably, Dosemeci et al. [16] concluded that repeat TCD examinations should be executed specifically in the setting of an initial TCD that demonstrates flow.

### Limitations

Our study consists of limitations that must be acknowledged. It is possible that selection bias may have led to the findings of our study. TCD was not the only ancillary test available during the study period; the choice was left to the attending providers. We had 17 patients who had circulatory arrest on the first study but did not have a second study. We tried to account for any selection bias by comparing that group to the final study population and found no significant differences. The TCD technicians and evaluators were not blinded to the TCD examinations in our study because of the retrospective clinical design. The power of our study was limited because of our small final sample size of 48 patients. There was also no gold standard available in our study for the included patients to assess the sensitivity and specificity of repeat TCDs. Lastly, our study contained limitations inherent to retrospective design (e.g., potential for residual confounding, incomplete or inaccurate chart data, or misclassification of exposures or outcomes).

## Conclusions

Our findings do not support current recommendations of two TCDs as ancillary testing for BD determination. In no instance did the second TCD show cerebral flow when the first TCD examination was consistent with neurocirculatory arrest. Hospital resources may be more effectively and efficiently allocated to optimize patient care and systemic institutional infrastructure. Further investigation and studies such as ours in larger populations are warranted to sufficiently support this conviction.
